# Natural history and clinical significance of aortic focal intimal flaps

**DOI:** 10.3389/fcvm.2022.959517

**Published:** 2022-10-04

**Authors:** Austin Maas, Pieter A. J. van Bakel, Yunus Ahmed, Himanshu J. Patel, Nicholas S. Burris

**Affiliations:** ^1^Department of Radiology, University of Michigan, Ann Arbor, MI, United States; ^2^Department of Cardiac Surgery, University of Michigan, Ann Arbor, MI, United States; ^3^Department of Vascular Surgery, University Medical Center Utrecht, Utrecht, Netherlands

**Keywords:** aortic disease, intimal flap, atherosclerosis, penetrating atherosclerotic ulcer (PAU), aortic dissection

## Abstract

**Objective:**

Focal intimal flaps (FIF) are a variety of defects of the aorta that result in a short, flap-like projection into the lumen, and are often encountered in asymptomatic patients undergoing computed tomography angiography (CTA) surveillance for aortic aneurysm, but the natural history and clinical significance of such lesions has not yet been studied.

**Methods:**

We retrospectively identified patients with an asymptomatic FIF and available imaging follow-up (>1 year). FIF was defined as flap-like intimal irregularity < 4 cm in length involving the thoracic aorta (TA), abdominal aorta (AA) or common iliac arteries (CIA). FIF characteristics included length and circumferential extent as well as the presence and size (width and depth) of associated penetrating aortic ulcers (PAUs). Patient characteristics, adverse events and history of surgical repair was determined by chart review. FIFs and associated PAUs were assessed for progression by comparing baseline and follow-up CTA studies.

**Results:**

A total of 84 FIFs were identified in 77 patients. Average age was 69.2 ± 10.1 years, and 81% were male (81%). Common co-morbidities included: hypertension (78%), hyperlipidemia (68%), smoking (60%), coronary artery disease (41%), aortic aneurysm (34%), type II diabetes mellitus (27%) and prior cardiovascular surgery (25%). FIFs were most commonly located in the abdominal aorta (*n* = 50, 60%). Nearly all FIFs were associated with local atherosclerotic plaque (93%). Mean follow-up interval was 3.5 ± 2.6 years (259 cumulative follow-up years). Change in FIF length and local aortic diameter over follow-up were 0.7 ± 2.3 mm and 0.8 ± 1.1 mm, respectively. Nearly half (47%) of FIFs were associated with penetrating aortic ulcers (PAU) with baseline depth of 7.3 mm (IQR: 6.1–10.2) and change in depth of 0.5 ± 1.4 mm. Only 12% of FIFs and 0% of associated PAUs demonstrated growth (≥3 mm) at follow-up. No acute pathology developed in the location of FIFs and no aortic interventions were performed specifically to treat FIFs.

**Conclusion:**

Focal intimal flaps identified in asymptomatic patients with aortic disease were co-localized with atherosclerotic plaque and PAUs, and demonstrated indolent behavior, not leading to significant growth or acute aortic events, supporting a conservative management approach.

## Introduction

Acute aortic syndrome (AAS) describes a variety of painful and potentially lethal acute aortic abnormalities that require accurate and timely diagnosis and treatment (1). Classic aortic dissection (AD) is the most common cause of AAS and is typically diagnosed by computed tomography angiography (CTA), characterized by the presence of an intimal flap and a contrast-opacified false lumen (2). A less common but increasingly recognized variant of classic dissection (∼5% of AAS cases) is termed limited intimal tears (LIT), which is characterized localized bulging of the aortic wall due to partial thickness tearing, often with an associated short-segmental intimal flap, although without false lumen formation (3, 4). Such LIT lesions are typically managed according to the Stanford classification of aortic dissections (5). Furthermore, in the setting of traumatic injury, small intimal flaps can be an indication of minimal aortic injury (MIA) (6).

While classic AD, LIT and MIA are all acute pathologies characterized by intimal disruption and flap formation, similar small intimal flaps are after often identified incidentally in the thoracic and abdominal aorta among asymptomatic patients undergoing imaging surveillance for chronic aortic pathology such as aneurysm or penetrating aortic ulcer (PAU). To date there are no published studies focused specifically on small intimal flaps in asymptomatic patients and the natural history and clinical significance of such abnormalities is poorly understood. Given this lack of evidence, clinical management approaches can be highly variable ranging from being ignored to being treated as chronic dissections and surveilled by regular imaging (7). A recent study of 273 patients with asymptomatic PAUs undergoing imaging surveillance described a very low rate of growth or complications with such lesions (8), although a study of 315 patients with PAU including those symptomatic lesions described a comparatively higher rate of complications and surgical repair (9). However, this study did not specifically describe lesions on the intimal surface of the aorta with a flap-like appearance, a morphology which can invoke a diagnosis of aortic dissection. Thus, there is a significant need for a systematic evaluation of small intimal flaps in an asymptomatic patient population to better determine the natural history and clinical significance of these lesions. The objective of this study was to identify patients with asymptomatic small intimal flaps and available serial CTA imaging to determine associated patient characteristics, quantify the degree of change in their dimensions, and defined the incidence of complications during long-term follow-up.

## Materials and methods

### Ethical statement

This retrospective study was performed as part of an Institutional Review Board (IRB), HIPAA-compliant study (HUM00159928, approved 03-27-2019) with a waiver of informed consent at the University of Michigan, Ann Arbor, Michigan.

### Study population

We performed a retrospective analysis of imaging and electronic medical record data from all patients that were diagnosed with an asymptomatic focal intimal flap (FIF) between January 2000 and January 2020. FIF was defined as flap-like intimal irregularity involving the thoracic aorta (TA), abdominal aorta (AA) or common iliac arteries (CIA), without associated intramural hematoma or classic dissection. The University of Michigan’s Electronic Medical Record Search Engine (EMERSE) was used to perform free text search of diagnostic reports of computed tomography angiography (CTA) or magnetic resonance angiography (MRA) reports mentioning a variety of terms that would raise possibility of FIF (10). The following search terms were used: “short segment flap”; “limited intimal flap”; “short flap”; “limited dissection”; “focal dissection flap”; “intimal irregularity”; “flap like”; “short segment dissection”; “penetrating atherosclerotic ulcer” (PAU); “penetrating aortic ulcer”; “ulcer like projection”; “discrete dissection”; or “subtle dissection.” Patients who were identified by one of the above search terms and also had ≥ 2 CTA or MRA studies available for analysis were included. Exclusion criteria included: longest imaging surveillance interval of <1 year; no evidence of FIF (as defined above); symptomatology consistent with acute aortic syndrome; FIF location distal of the common iliac artery (CIA); poor imaging quality; imaging findings of IMH or classic aortic dissection on imaging; or FIF located ≤ 2 cm from aortic aneurysm or sites of surgical repair. The earliest available contrast enhanced CTA/MRA in which the FIF was identified was used for baseline measurements and the most recent available scan was analyzed to determine change in FIF morphologic parameters during surveillance.

### Clinical characteristics

Patient demographics, clinical history and outcomes were collected by chart review. Demographics variables included age and sex. Clinical variables included history of hypertension, hyperlipidemia, smoking, diabetes mellitus and atherosclerotic disease. Outcome variables were extracted from the electronic medical record and included development of aortic related symptoms, development of classic aortic dissection or IMH at the site of FIF, and open or endovascular surgical repair related to the FIF during follow-up.

### Imaging characteristics

All CT or MRI examinations were reviewed by a medical student (AM) and an experienced cardiovascular radiologist (NB) to select patients with FIFs within any portion of the thoracic aorta, abdominal aorta or common iliac arteries. FIFs were defined as small intimal irregularities of the luminal surface of the aorta resulting in a flap-like projection into the aortic lumen, measuring < 4 cm in length, and without associated imaging findings of classic intramural hematomas (IMH) or aortic dissection.

**FIGURE 1:**
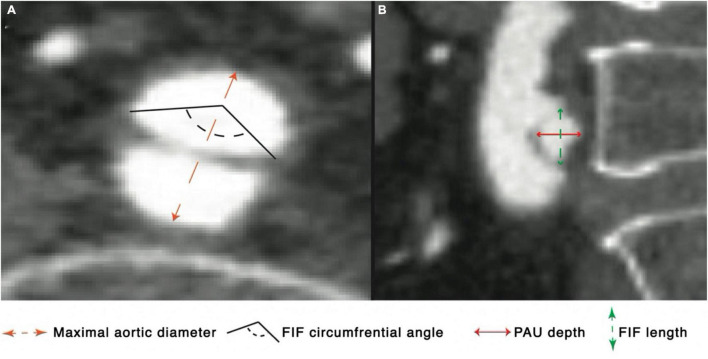
Diagram depicting measurements performed on axial **(A)** and longitudinal **(B)** plane using CTA in a patient with a PAU-like focal intimal flaps (FIF).

The following measurements were obtained for each FIF on the initial and most recent scans available: longitudinal length, circumferential involvement extent (in degrees), depth, maximal and minimal aortic diameter at the location of FIF (Figure 1). The presence of macroscopic atherosclerotic plaque at the location of each FIF was recorded, defined as non-calcified or calcified plaque at the aortic intima within 1 cm from the FIF. Patients with FIF were further subclassified into groups based on the presence of absence of an associated PAU (PAU-like FIF), with PAU defined as an irregular outpouching of contrast into the wall of the aorta with associated outward bulging of the adventitial contour. FIF depth was only calculated for PAU-like lesions. The location of each FIF was documented as aortic root, ascending aorta, aortic arch, descending thoracic aorta, abdominal aorta, or CIA. FIFs were characterized as growing or stable; growth being defined as an increase in any FIF dimension by ≥ 3 mm.

### Statistical analysis

Categorical variables were reported as frequencies and percentages and analyzed using univariate analysis, such as the use of Chi-square test. Continuous variables were reported as mean (SD) or median (interquartile range) depending on their normality. Student’s *T*-test or Mann–Whitney U-test were used to analyze continuous variables. A *P*-value of <0.05 was considered statistically significant. Data analysis was performed using IBM SPSS Statistics for Windows, Version 25.0 (IBM Corp., Armonk, NY, USA).

## Results

### Patient characteristics and follow-up

**FIGURE 2:**
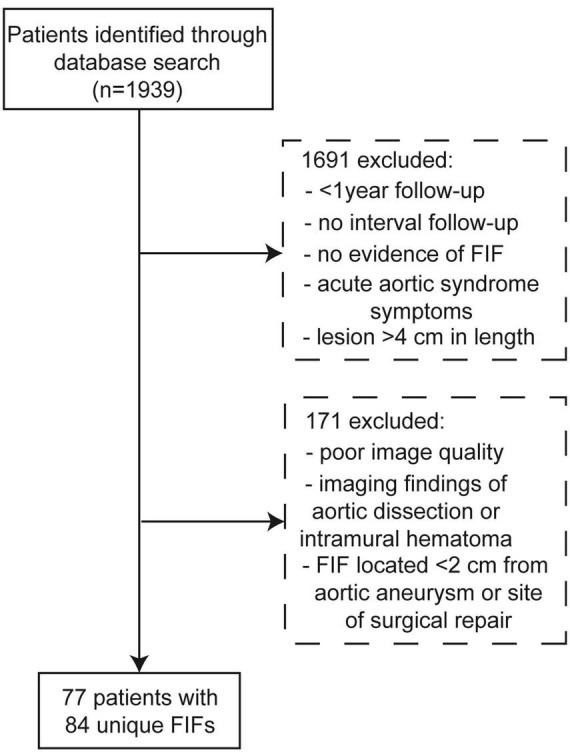
Patient selection flowchart.

After application of the inclusion and exclusion criteria, 77 patients were deemed appropriate for analysis with 84 unique FIFs identified (Figure 2). The mean age was 69.2 ± 10.1 years old at diagnosis and the majority of patients was male (80.5%, 62/77). Mean BMI was 29.1 ± 4.8 kg/m^2^. The mean follow-up time was 3.4 ± 2.6 years with a cumulative 259 follow-up years. Cardiovascular risk factors were common among the study population with 77.9% (60/77) of patients having a history of hypertension and over half had a history of smoking (59.8%; 46/77); among smokers the average pack-years were 42.3 ± 32.4 years. The majority of patients had known the aorta or its principal branches at the time of FIF discovery (64%, 49/77) with primary indications for CTA imaging including: abdominal aortic aneurysm (*n* = 8, 10%); root/ascending aortic aneurysm (*n* = 16, 21%); giant cell aortitis (*n* = 1, 1%); thoracoabdominal aortic aneurysm (*n* = 2, 3%); repaired DeBakey type II dissection (*n* = 2, 3%); penetrating aortic ulcer (*n* = 12, 16%); aortoiliac occlusive disease (*n* = 4, 5%); aberrant right subclavian artery with diverticulum of Kommerell (*n* = 1, 1%); carotid artery occlusion (*n* = 1, 1%). The most common aortic disease was aortic aneurysm (34%, 26/76), and approximately a quarter of patients (25%, 19/77) had undergone prior aortic interventions in aortic segments outside of the location of FIF prior to baseline imaging. Complete clinical and demographic data are included in Table 1.

**TABLE 1:** Patient characteristics.

Patient characteristics				

Characteristic	Overall (*n* = 77)	PAU	No-PAU	*P*-value
Age (y)	69.2 ± 10.1	70 ± 9.2	69 ± 11.0	0.820
Male sex, n (%)	62 (80.5)	31 (79.5)	31 (81.6)	0.817
BMI (kg/m2), mean ± SD	29.1 ± 4.8	28.5 ± 4.7	29.7 ± 4.9	0.289
Hypertension, n (%)	60 (77.9)	28 (73.7)	32 (82.1)	0.376
Diabetes Mellitus, n (%)	21 (27.3)	5 (13.2)	16 (42.1)	0.005
Hyperlipidemia (%)	52 (68.4)	27 (71.1)	25 (65.8)	0.622
Tobacco use, n (%)	46 (59.8)	26 (66.6)	20 (52.7)	0.209
Average pack years, mean ± SD	42.3 ± 32.4	47.6 ± 41.5	38.2 ± 23.2	0.364
COPD, n (%)	9 (11.8)	3 (3.9)	6 (15.8)	0.287
CAD, n (%)	31 (40.8)	12 (31.6)	19 (50.0)	0.102
Prior MI, n (%)	12 (15.8)	4 (10.5)	8 (21.7)	0.208
Prior stenting, n (%)	13 (16.9)	4 (10.5)	9 (23.7)	0.128
Prior cardiovascular surgery, n (%)	19 (25.0)	8 (21.1)	11 (28.9)	0.427
Chronic Kidney Disease, n (%)	6 (7.8)	2 (5.3)	4 (10.5)	0.395
Prior stroke, n (%)	9 (11.8)	3 (7.9)	6 (15.8)	0.287
Peripheral vascular disease, n (%)	8 (10.4)	1 (2.6)	7 (18.4)	0.025
Connective tissue disease, n (%)	1 (1.3)	0 (0.0)	1 (2.6)	0.314
Family history of aortic disease, n (%)	3 (3.9)	3 (7.9)	0 (0.0)	0.077
History of heart failure, n (%)	4 (5.3)	1 (2.6)	3 (7.9)	0.304

### Imaging features

**FIGURE 3:**
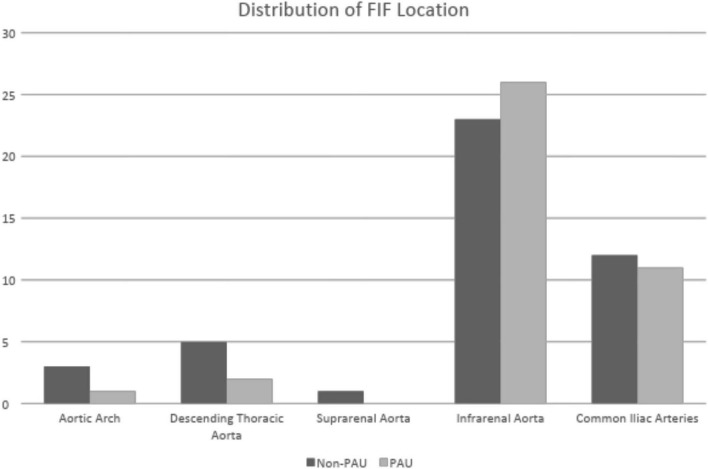
Histogram of FIF anatomic location by aortic segment.

**FIGURE 4:**
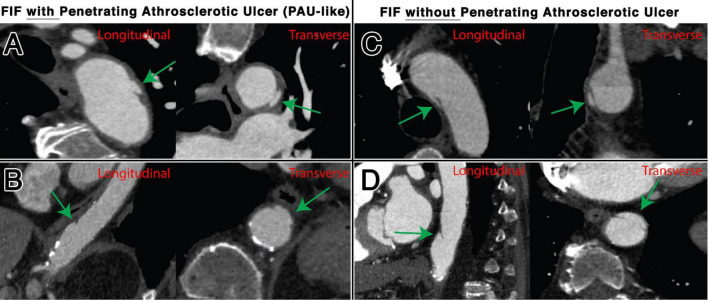
Representative examples of FIFs, with and without associated PAU, located in the thoracic aorta, shown transverse and longitudinal plane. Locations and characteristics of FIFs shown include distal aortic arch with PAU **(A)**, distal descending thoracic aorta with PAU **(B)**, mid arch without PAU **(C)**, and distal descending thoracic aorta without PAU **(D)**.

**FIGURE 5:**
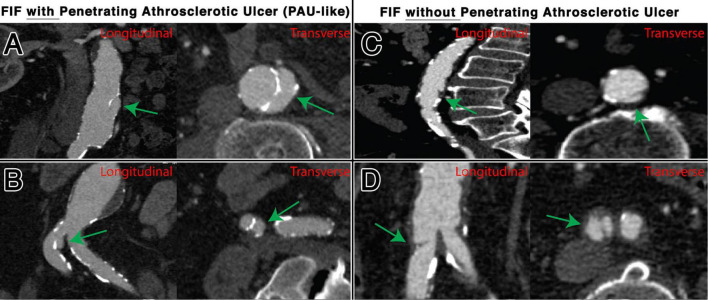
Representative examples of FIFs, with and without associated PAU, located in the abdominal aorta, shown transverse and longitudinal plane. Locations and characteristics of FIFs shown include infrarenal segment with PAU **(A)**, common iliac artery with PAU **(B)**, infrarenal segment without PAU **(C)**, and common iliac artery without PAU **(D)**.

Focal intimal flaps were most frequently located in the abdominal aorta (59.5%, 50/84), with the second most common location being the CIAs (27.3%, 23/84) (Figure 3). Imaging variables at baseline, as detailed in Table 2, revealed an overall median FIF length of 14.0 mm (IQR: 10.4–20.1). The median aortic diameter at the location of the FIFs was 21.6 mm (IQR: 18.2–25.4). The majority of FIFs were associated with localized (within 1 cm of FIF) atherosclerotic plaque (93%, 78/84), with localized plaque characterized as: absent (7%, 6/84), non-calcified only (10%, 8/84), calcified only (7%, 6/84), and mixed calcified and non-calcified plaque (76%, 64/84). PAU-like lesions were common (47%, 40/84), with an average PAU depth of 7.3 mm (IQR: 6.1–10.2). Representative images of FIFs are displayed in Figures 4, 5.

**TABLE 2:** Focal intimal flaps (FIF) imaging characteristics.

FIF imaging characteristics	

Characteristic	Overall (*n* = 84)
**Location**	
Aortic Arch, n (%)	5 (5.9)
Descending Thoracic Aorta, n (%)	7 (8.3)
Abdominal Aorta, n (%)	50 (59.5)
Common Iliac Artery, n (%)	23 (27.3)
**FIF Dimensions**	
Length (mm), median (IQR)	14.0 (10.4–20.1)
Circumferential Angle (degrees), median (IQR)	108.0 (92.6–128.0)
Maximum Aortic Diameter (mm), median (IQR)	21.6 (18.2–25.4)
Minimum Aortic Diameter (mm), median (IQR)	19.9 (16.2–22.9)
Associated with PAU, n (%)	40 (47)
PAU Depth (mm), median (IQR)	7.3 (6.1–10.2)
Associated with Atherosclerosis, n (%)	79 (94)

### Outcomes

The mean change in length of FIFs was 0.65 ± 2.3 mm, with a maximum change of 9.7 mm (a 46% increase). Seventy-six (90.5%) FIFs were stable over imaging follow-up (stability defined as < 3 mm of change in any dimension). The mean change in maximal aortic diameter at the level of the FIF was 0.8 ± 1.1 mm with a maximum change of 2.9 mm. Mean change in PAU depth was 0.5 ± 1.4 mm. The mean aortic growth rate at the location of the FIF was 0.4 ± 0.6 mm/year and did not differ between FIFs with and without associated PAU (0.4 ± 0.6 versus 0.3 ± 0.5, *p* = 0.731). While 8 (9.4%) patients developed symptoms of arterial occlusive disease during follow-up (*n* = 6 lower extremity claudication, *n* = 1 abdominal claudication), no patients developed acute symptoms that we clinically attributed to the FIF (or aortic segment containing the FIF). There were no FIFs associated with other outcomes, such as aortic dissection, intramural hematoma, aortic rupture, or aorta-related mortality. Ten (12.9%) patients underwent unrelated aortic procedures during FIF follow up; one procedure involved an FIF due to an extensive abdominal aortic aneurysm repair. Patient outcomes during follow-up can be found in Table 3.

**TABLE 3:** Patient outcomes.

Patient outcomes	

**Outcome**	**Overall**
Mean change in lengthd (mm ± SD)	0.65 ± 2.3
Stable FIF, n (%)	76 (90.5)
Growing FIF, n (%)	8 (9.5)
Mean change in aortic diameter	0.8 ± 1.1
Aortic-related symptoms, n (%)	0 (0)
Aortic procedure during follow-up	10 (12.9)
Procedure involving FIF, n (%)	1 (1.2)

## Discussion

The objective of this study was to examine the natural history of small aortic intimal flaps (FIFs) among a cohort of asymptomatic patients undergoing imaging surveillance with CTA/MRA. We were able to examine baseline clinical characteristics and imaging findings of asymptomatic FIF with long-term follow-up (cumulative of 259 person-years) of clinical and radiological outcomes. Key findings of our study can be summarized as follows: (i) no patients developed dissection/rupture, significant aortic growth or aorta-related complication at the location of the FIF, (ii) the vast majority of FIFs (91%) demonstrated no measurable change during follow-up, (iii) the main location for FIF in asymptomatic patients was the abdominal aorta, (iv) almost half of FIFs (47%) were associated with PAU-like lesions. The present study is unique in that it observes the natural history of FIFs in asymptomatic patients, unlike prior studies which have focused on the clinical significance and treatment of small intimal flap-like abnormalities among symptomatic patients.

The majority of patients with FIFs in this study had multiple cardiovascular risk factors commonly associated with aortic disease such as male sex, hypertension, hyperlipidemia and a history of smoking. Additionally, the majority of these patients also had pre-existing aortic disease, most commonly aortic aneurysms. Interestingly, the vast majority of FIFs were associated with atherosclerotic plaque at the location of the lesion and the majority of FIF in this study were located below the diaphragm (abdominal aorta and common iliac arteries), which is not unexpected given the predilection of atherosclerosis for the abdominal aorta (11, 12). While there were a small number of FIFs (*n* = 6) in our cohort which occurred in the absence of any identifiable atherosclerotic plaque by CT, the co-localization of these lesions with the presence of plaque supports the concept that such flap-like intimal abnormalities are related to the inflammatory and erosive processes that lead to plaque ulceration and ultimately a PAU (13). Additionally, hemodynamic differences between the thoracic and abdominal aorta could play a role the predilection of FIFs for the abdominal aorta (11, 14). Finally, supporting the strong association between FIF and atherosclerosis over more acute pathologies (e.g., dissection, intramural hematoma) is the observation that FIFs were found to be indolent lesions, demonstrating little to no change over years of imaging follow-up and resulting in no adverse events. Our results mirror previously published studies focused on the natural history and outcomes associated with small asymptomatic PAUs, in which that similarly found these lesions to be indolent and associated with a low rate of growth or development of adverse events (8, 15–17). While atherosclerosis likely plays some role in the development of such FIF lesions, other etiologies have been described, such as residua of prior IMH (18, 19). While patients with IMH a documented history of IMH, dissection or acute aortic syndrome were excluded from this study, and there were no imaging findings of IMH in the CTAs analyzed, given the age and comorbidities observed in this cohort its plausible that other etiologies including sequala of a prior dissection or IMH may contribute to the formation of some FIF lesions.

Due to increased availability and use of high-resolution CT/MR imaging to monitor aortic disease (20), it seems reasonable to expect the incidental identification of asymptomatic FIFs by medical imaging to increase in the future. A consequence of increased detection could mean that a growing number of patients may be subjected to lifelong imaging surveillance for FIF, even in the absence of a separate aortic pathology that would warrant surveillance (e.g., PAU, aneurysm, dissection). Based on the results of our study, frequent imaging surveillance dedicated to these small intimal abnormalities appears unnecessary in the absence of PAU or other significant aortic pathology. Beyond the general description of the presence and degree of atherosclerotic plaque, targeted descriptions and measurements of FIFs in diagnostic imaging reports may perpetuate a tendency to perform dedicated surveillance of such lesions. Further research is needed to determine the need for lifelong imaging surveillance and the optimal frequency of follow-up imaging.

### Limitations

There are several limitations concerning the results of this study. First, there are inherent limitations to the retrospective, single-center nature of our data collection, with inability to accurately capture some variables with high fidelity (e.g., patient reported symptoms, remote histories of trauma or arterial catheterization) and unclear generalizability of our findings to other populations with different demographics and risk-factors. Additionally, there is likely selection bias in our sample as patients with more or less severe aortic disease may not be undergoing routine imaging surveillance and thus not included in our cohort, and there is no control group of patients with similar risk factors but without indications for CTA imaging. Secondly, there are inherent limitation in the free-text search tools methods that we used to identify FIFs in CT reports, as there are likely many FIFs present in imaging exams at our institution which were not captured in our study if they were not commented on specifically in the diagnostic report. Finally, we only included patients with follow-up imaging given our desire the determine the long-term changes in FIFs, however, limiting our study to patients with longitudinal imaging may have biased us toward a population with more severe manifestations of aortic disease, although increased inclusion of low-risk patients would be unlikely to significantly change our primary findings.

## Conclusion

Overall, our results suggest that FIFs are largely indolent lesions associated with chronic aortic disease of the descending thoracoabdominal aorta in asymptomatic patients. The vast majority of FIFs demonstrate little or no change over longitudinal follow-up and we did not observe the development of dissection or IMH during follow-up in any cases. Thus, such small FIFs do not appear to warrant aggressive imaging surveillance in asymptomatic patients, especially when located in the abdominal aorta or common iliac arteries, and when not associated with PAU or aortic dilation.

## Data availability statement

The datasets presented in this article are not readily available because protected health information. Requests to access the datasets should be directed to NB, nburris@med.umich.edu.

## Ethics statement

The studies involving human participants were reviewed and approved by the University of Michigan IRB (HUM00159928). Written informed consent for participation was not required for this study in accordance with the national legislation and the institutional requirements.

## Author contributions

NB, PB, AM, HP, and YA contributed to conception and design of the study. AM, PB, YA, and NB organized the database and performed the statistical analysis. AM and PB wrote the first draft of the manuscript. YA and NB wrote sections of the manuscript. All authors contributed to manuscript revision, read, and approved the submitted version.
